# Awake Craniotomy for Resection of Intracranial Meningioma: First Case Series From a Low- and Middle-Income Country

**DOI:** 10.7759/cureus.18716

**Published:** 2021-10-12

**Authors:** Saqib Kamran Bakhshi, Noyan Jawed, Faraz Shafiq, Syed Ather Enam

**Affiliations:** 1 Neurosurgery, Aga Khan University Hospital, Karachi, PAK; 2 Surgery, Aga Khan University Hospital, Karachi, PAK; 3 Anaesthesiology, Aga Khan University Hospital, Karachi, PAK

**Keywords:** scalp block, tumors, meningioma, extra-axial lesions, awake craniotomy

## Abstract

Introduction

Awake craniotomy (AC) has emerged as a better modality for resection of intra-axial brain tumors. The advantages are not just related to the preservation of neurological function, but also include early recovery, short hospital stay and possibly lower costs. However, data on AC for meningioma resection is deficient, likely because of concerns related to intra-operative pain and blood loss.

Methods

All patients who underwent AC, using awake through-out technique for resection of meningioma, during the last five years, were included in the study. Non-probability consecutive sampling technique was employed. Variables for demographics, and details of diagnosis and surgical procedure were recorded. The outcomes measured were length of hospital stay, worsening of neurological function during surgery and significant intra-operative or post-operative pain.

Results

Seventeen patients underwent AC for resection of meningioma during the study period. Eleven of these were grade I meningioma, and six were grade II meningioma. The mean age was 45.8 ± 10.5 years. Presenting complaints were variable, with seizures being the most common (n = 7; 41.2%). The mean duration of surgery was 180.8 ± 36.2 minutes and median estimated blood loss was 450 ml (IQR: 225 ml - 737.5 ml). The mean length of stay in the hospital was 3.1 ± 1.3 days. Only one patient had a prolonged hospital stay of seven days, because of post-operative seizures. Eleven patients (58.3%) had convexity meningioma, 4 (33.3%) had parasagittal meningioma and 1 each had a parafalcine and anterior skull-base meningioma. Simpson grade I resection was performed in 6 (41.7%) patients, grade II resection in 10 (50%) patients, and grade III resection in 1 (5.9%) patient. None of our patients had deterioration in their neurological deficits after surgery and no one required emergency intubation, conversion of surgery to general anesthesia, or redo exploration.

Conclusion

AC may be considered a safe modality for surgical resection of convexity and parasagittal meningioma, with no significant risk of intra-operative or post-operative pain, although it requires more evidence. It can be offered to patients who are at higher risk, or are not willing to undergo general anesthesia. Ultimately, it might also be beneficial in terms of reducing overall costs.

## Introduction

Surgical resection of brain tumors under awake craniotomy (AC) has gained popularity during the last two decades [1]. The technique was initially introduced for epilepsy surgery more than two hundred years ago. Nowadays, it is frequently being opted for resection of tumors near eloquent cortex [2,3]. It is considered the gold standard tool for real-time localization of functionally important areas in the brain during surgery, thereby decreasing dependence on often inconsistent anatomic landmarks for brain mapping [4].

Literature review, however, suggests that the advantages of AC are not just limited to safe tumor resection. AC for brain tumor surgery not only allows for continuous monitoring of patients’ neurological function, but also saves them from the risks of general anesthesia. It results in saving resources in low- and middle-income countries by decreasing hospital length of stay and critical care admissions [5,6]. Recent data also suggests that AC has a significant role in promoting faster recovery, limiting the need for extensive post-operative care [7]. Although most of the published literature supports AC for resection of intra-axial brain tumors, there have been some evidence highlighting its importance for extra-axial tumors as well [8-10].

Meningiomas are the most common extra-axial brain tumors in the brain. These are dural-based lesions, and it has been an assumption that performing AC for meningioma would be associated with significant intra-operative pain on dural manipulation. There have been few case reports and case series, reporting successful AC for resection of meningioma and other extra-axial tumors [8-10]. Considering the dearth of literature on the subject, our objective was to report technique employed at our health care setup for resection of meningioma using AC.

## Materials and methods

Patient population

All patients who underwent AC for resection of meningioma between January 2016 to January 2021 were included. The detail of these patients was retrieved from operating room database. Ethical review board’s exemption was also taken. The sampling technique was non-probability consecutive sampling. Data was collected on a pre-designed data collection form, which had details about demographics, diagnosis and intraoperative and postoperative management. Statistical analysis was conducted using SPSS Inc. Version 21.0. Continuous data has been represented as means and standard deviation, while categorical data has been represented as frequencies and proportions. All cases were performed by the same surgeon (the senior author), and the same anesthetic technique was used to maintain uniformity and to avoid variability.

The outcomes included length of hospital stay, worsening of neurological examination during surgery, which was present in the neurological examination on first follow-up in clinic (10 days from surgery), and significant intra-operative or post-operative pain. Neurological worsening was defined as any impairment in patients’ function of speech, gross motor power in the limbs, sensations in the limbs, or vision in any eye at the time of clinic follow-up (10 days from surgery). This information was extracted from the patients’ pre-operative and first follow-up in clinic neurological examinations, mentioned in their medical record files. Since it was a retrospective review, pain was assessed according to the patients’ analgesic requirement during or within 24 hours after surgery. Extent of tumor resection was graded according to the Simpson grading system [11].

Surgical technique and anesthesia for AC

As per our routine practice, all patients planned for AC had initial preoperative assessment and counselling by neuro-anesthetist. The dedicated neuro-anesthesia team managed the perioperative care of these cases. It included protocol-based anesthetic care, comprising of handover of AC-related educational brochure to the patient, preoperative urinary catheterization on the night before surgery and scheduling of these cases as first on the list. Patients were also reassured about possible discomforts like bone drilling and feeling of pain during dural retraction. An awake throughout the approach of AC was employed for all study patients. This included institution of scalp block and providing conscious sedation using dexmedetomidine infusion. There were no absolute contraindications to Dexmedetomidine, however, patients were observed for possible adverse effects including bradycardia, hypotension and hypertension. For scalp block, Ropivacaine 0.5 % (30-40 ml) along with adrenaline and 8 mg dexamethasone was used.

Routine ASA (American Society of Anesthesiologists) specified monitoring was done during surgery, including heart rate, non-invasive/invasive blood pressure, saturation and end-tidal CO2. The depth of sedation was titrated according to Bispectral Index (BIS) monitoring, level of which was kept between 75-80 during tumor resection phase. Additional doses of fentanyl were occasionally required during dural retraction. We gave Oxygen to all patients via nasal prongs at 2 L/min, which has got in-built end-tidal CO2 monitoring port. Patients were also administered intravenous Paracetamol 1 gm, at the start of operation. In all cases, once craniotomy was done, 2% xylocaine with adrenaline-soaked gauze was placed on the dura for 1 minute for local absorption, before durotomy. Double antiemetic prophylaxis was given using dexamethasone and ondansetron (0.1 mg/kg). Levetiracetam 1 gm stat was prescribed for perioperative seizure prophylaxis. All procedures were performed under microscope, and Cavitron ultrasonic aspirator (CUSA) was also used in some cases for tumor resection. Most of the patients were not administered Mannitol at the beginning or during surgery.

Post-operative management

Post-operative management included the transfer of patient to post-anaesthesia care unit and then to a high dependency unit for overnight observation. Postoperative pain management was done with intermittent doses of Paracetamol 1 gm Q6H, and tramadol 50-60 mg Q 6-8 hourly. Antiemetic and Seizure prophylaxis was also continued for 48 hours postoperatively.

## Results

Seventeen patients underwent AC for resection of extra-axial lesions during the study period, all of which were later diagnosed to have meningioma on histopathology. Most of the patients were young, with the mean age of these patients being 45.8 ± 10.5 years. Presenting complains were variable, with seizures being the most common (n = 7; 41.2%). The mean duration of surgery was 180.8 ± 36.2 minutes and the median estimated blood loss (EBL) was 450 ml (IQR: 225 ml - 737.5 ml). The mean length of stay in the hospital was 3.1 ± 1.3 days. Only one patient had a prolonged hospital stay of seven days because he had developed seizures which were controlled by escalating antiepileptic drug dosage. Details of demographics are shown in Table 1.

**Table 1:** Demographics. HTN: hypertension; IHD: ischemic heart disease; DM: diabetes mellitus.

Case	Gender	Age (years)	Co-morbids	Presenting complains	Duration of surgery (mins)	Estimated blood loss (ml)	Length of hospital stay (days)
1	Male	25	None	Seizure	150	750	3
2	Male	45	None	Seizure	215	800	3
3	Male	41	HTN	Impaired sensory function	160	200	3
4	Male	35	None	Impaired sensory function	230	1500	3
5	Female	30	None	Visual impairment	173	700	3
6	Female	48	None	Impaired motor function	201	350	2
7	Male	49	None	Seizure	125	50	2
8	Male	55	None	Seizure & Impaired motor function	175	500	4
9	Male	39	IHD	Headache	140	200	2
10	Female	42	HTN	Speech impairment	217	400	3
11	Male	50	HTN & DM	Impaired sensory function	155	500	7
12	Male	47	None	Impaired motor function	165	300	2
13	Male	62	HTN	Seizure	213	600	3
14	Male	48	None	Seizure	125	250	3
15	Female	56	None	Visual impairment	240	1150	5
16	Female	65	HTN	Speech impairment	210	900	2
17	Male	42	DM	Seizure	180	100	3

**Figure 1:**
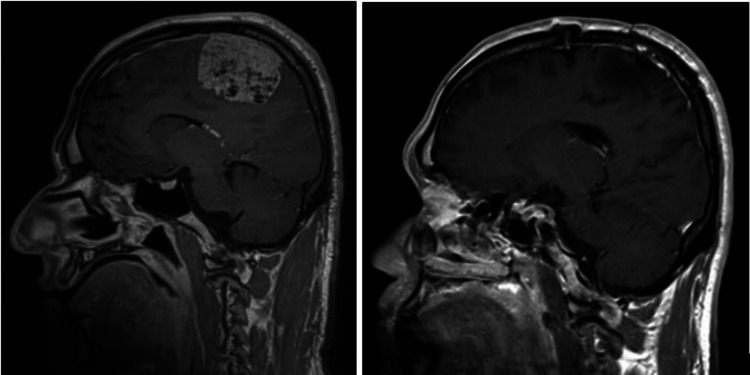
MRI brain T1 post-contrast, sagittal section, pre-operative (left) and post-operative (right) images. The parasagittal meningioma was located in the middle of the superior sagittal sinus, and a small part of the lesion adherent to the sinus wall was left behind.

**Figure 2:**
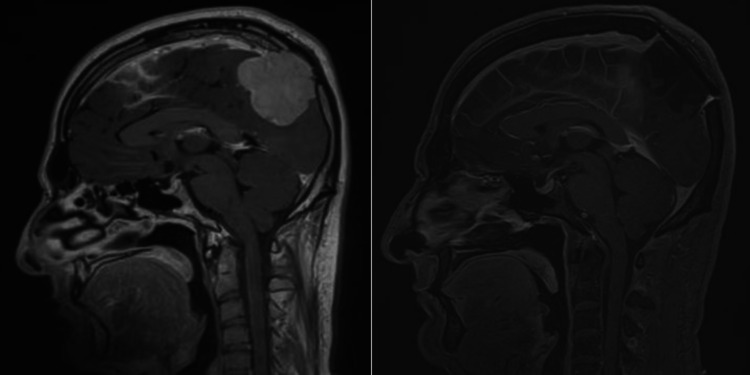
MRI brain T1 post-contrast, sagittal section, pre-operative (left) and post-operative (right) images. The posteriorly located parasagittal meningioma had thrombosed superior sagittal sinus, and was completely resected with duroplasty and cranioplasty with titanium mesh as seen in post-operative scan on the right.

**Figure 3:**
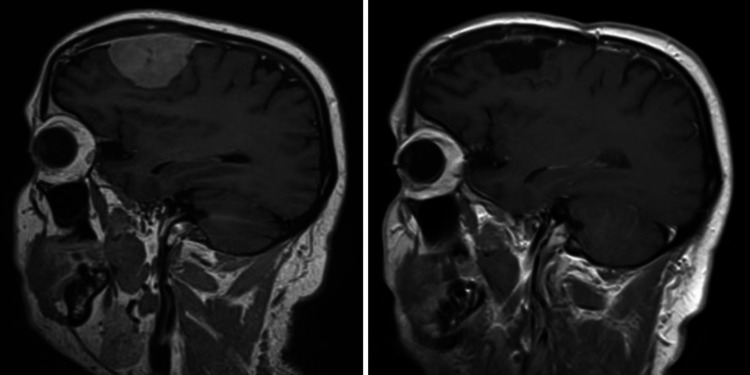
MRI brain T1 post-contrast, sagittal section, pre-operative (left) and post-operative (right) images. The frontal convexity meningioma was completely resected as seen in post-operative scan on the right.

**Figure 4:**
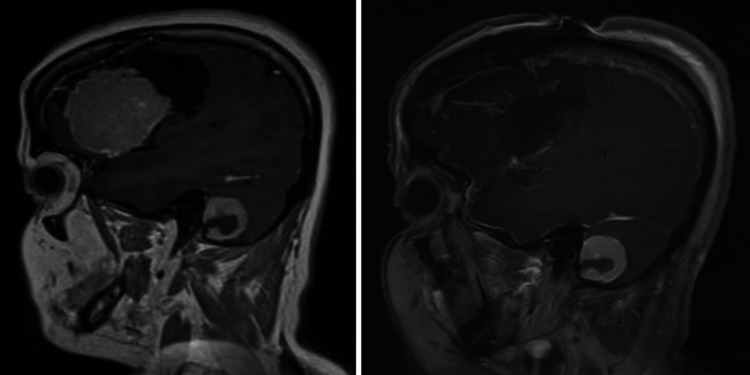
MRI brain T1 post-contrast, sagittal section, pre-operative (left) and post-operative (right) images. The lateral sphenoid-wing meningioma was completely resected as seen in post-operative scan on the right. Another homogenously enhancing lesion with a small hypo-intense central part, can be seen in the posterior fossa, which was not causing symptoms, was not resected.

Only one patient had the lesion located at skull-base (lateral sphenoid-wing); more than half of the patients (64.7%) had their lesions located at the convexity. Figures 1 to 4 are pre-operative and post-operative MRIs of some of the cases.

**Figure 5:**
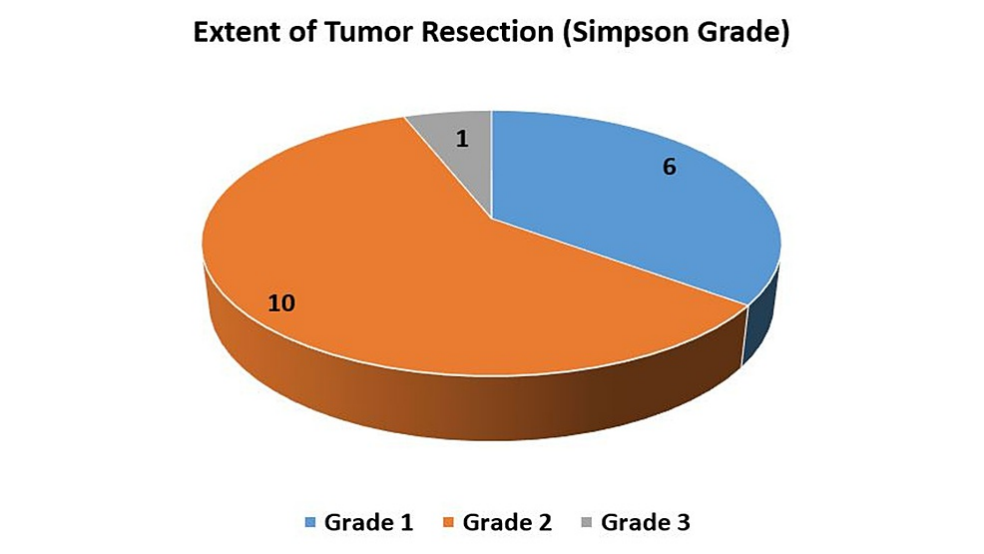
Extent of tumor resection according to Simpson grading.

Nine patients (52.9%) showed improvement in their functional status at follow-up. Two patients (case numbers 8 and 12) had a significantly poor functional status before surgery because of right hemiparesis which remained stable after surgery in one case and improved in the other. None of the patients required additional oral or intravenous analgesics, apart from the standard regime. All patients had received prophylactic antiemetics post-operatively (intravenous ondansetron 8 mg Q8H or intravenous metoclopramide 8 mg Q8H) for 48 hours, and none of them reported nausea or vomiting during or after surgery. The details of surgery and functional outcome are described in Table 2, and the extent of tumor resection is depicted in Figure 5.

**Table 2:** Details of diagnosis and surgery. KPS: Karnofsky performance score.

Case	Histopathological diagnosis	Site of Lesion	Side of Lesion	Extent of Resection (Simpson grade)	Admission KPS	KPS at last follow-up	Post-op changes in functional status
1	Meningioma grade II	Convexity	Right	2	90	90	No change
2	Meningioma grade II	Parasagittal	Left	2	70	90	Improvement
3	Meningioma grade II	Convexity	Left	2	90	100	Improvement
4	Meningioma grade I	Parasagittal	Left	1	80	100	Improvement
5	Meningioma grade I	Convexity	Right	2	90	100	Improvement
6	Meningioma grade I	Convexity	Left	1	70	80	Improvement
7	Meningioma grade II	Convexity	Right	1	90	100	Improvement
8	Meningioma grade II	Parasagittal	Right	1	50	50	No change
9	Meningioma grade I	Convexity	Right	1	90	100	Improvement
10	Meningioma grade I	Sphenoid wing	Left	2	80	100	Improvement
11	Meningioma grade I	Convexity	Right	3	90	90	No change
12	Meningioma grade II	Parasagittal	Left	2	60	70	Improvement
13	Meningioma grade I	Falcine	Right	2	80	80	No change
14	Meningioma grade I	Convexity	Left	2	90	90	No change
15	Meningioma grade I	Convexity	Left	2	80	80	No change
16	Meningioma grade I	Convexity	Left	2	80	80	No change
17	Meningioma grade I	Convexity	Right	1	90	90	No change

## Discussion

AC has been mainly employed in neuro-oncology for resection of intra-axial brain tumors. Coupled with cortical mapping, it has proved to be an important tool for the removal of tumors located in eloquent regions, particularly low-grade gliomas, with less surgical morbidity [12]. It allows a better chance of preservation of neurological function, notwithstanding the lesion’s size, primary pathology or patients’ history [13,14]. Lately, it has been shown in studies that AC can be utilized for resection of most supratentorial lesions including extra-axial tumors, cerebral abscess and arterio-venous malformations [15-17]. Many neurosurgeons now recommend AC to avoid risks associated with general anesthesia, and to decrease complication rates. Surveys have also revealed increased patient satisfaction when they undergo awake cranial surgery [17-19]. The only absolute contraindication to AC in literature is patient refusal, as compiled by Kaiying et al [20]. They had also reported mental health disorders, obstructive sleep apnea, inability to lay still for long periods, highly vascular large lesions and tumors at skull-base as relative contraindications to AC [20].

Although AC has been in practice for a long time, recent advancements in neuro-anesthesia and pain management have caused a resurgence and increased acceptance of this modality for neurosurgeons, as well as the patients [21]. Serlatis et al. had published a large prospective series of 610 patients who had undergone AC for supratentorial lesions, irrespective of the histopathology [5]. Eleven (1.8%) patients in their data had successful uneventful meningioma resection, and they had proposed this technique for resection of all kinds of intracranial lesions [5]. In a case report, Kumar et al. reported a 29 years old, 13 weeks pregnant patient who successfully underwent awake resection of a large meningioma [9]. In another case report, a 72 years old male patient had successfully undergone AC, far lateral approach, for resection of foramen magnum meningioma, with the patient in a three-quarter prone position [8].

A possible reason for underutilization of AC for excision of dural-based lesions is the fear of inadequate pain control. During surgery, the steps of bone flap elevation, durotomy and separation of tumor from dura or dural coagulation are the most painful steps. Inadequate pain relief at these steps can potentially result in the patient becoming restless and agitated, and might cause insufficient tumor resection [5]. Earlier studies had labelled lesions with significant dural involvement and high vascularity to be contraindications for AC [22,23]. The protocol-based anesthetic and surgical care worked very well for our study population. The fact that 50% of our patients underwent Simpson grade II resections where dura was coagulated, with no adverse event during surgery, strongly advocates for the use of this technique for resection of extra-axial tumors. Although the median EBL was 450 ml, five patients had an EBL of more than 700 ml which was managed with intra-operative blood transfusion and did not pose any significant challenge intra-operatively.

Another major limitation that should be considered while planning an AC, is the risk of emergency intubation to convert the procedure to general anesthesia, in patients who develop severe pain, threatened airway or seizures. None of our patients required conversion of the procedure to general anesthesia, although there is a 16% risk reported in the literature [24]. This might be related to the effective use of scalp block and BIS-guided sedation using Dexmedetomidine. There is around 5% risk of intra-operative seizures during AC, more with resection of intra-axial lesions, and may even result from cortical stimulation [5]. None of the patients in our series had intra-operative seizures, and only one patient had post-operative seizures that prolonged his hospital stay.

Studies have also reported less occurrence of post-operative nausea and vomiting after AC as compared to patients who receive general anesthesia [25]. We had prescribed intravenous ondansetron and dexamethasone to all of our patients post-operatively, so that may be a reason for no significant nausea and vomiting reported by our patients. One patient (5.9%) in our study had post-operative seizures. None of our patients developed post-operative surgical site hematoma, CSF leak or required re-operation or re-admission after discharge. Our complication rate is much less than that reported in the literature (approximately 15%), mostly for intra-axial tumors [5]. However, because of smaller number of cases, and the absence of comparison group, we recommend large cohort studies comparing AC and general anesthesia for meningioma resection, to draw more generally acceptable results.

## Conclusions

Based on the results from this case series, and the surgical technique described, we propose that AC may be safely used for resection of extra-axial tumors, particularly meningioma located at convexity or parasagittal sites, and intraoperative pain and blood loss are not a major concern. It can be offered as a possible alternate to patients who are afraid, unwilling or high risk to undergo general anesthesia. Even though cortical mapping is not of much benefit for these lesions, AC does prevent complications that can arise from general anesthesia, and can lead to early discharge from the hospital in most cases. More evidence is, however, needed to substantiate our findings, and to study the possibly lower costs of treatment and shorter hospital stay as compared to resection under GA.
